# Vehicle-to-Vehicle Secure Communication Protocol Based on Digital Vehicle Identification Number

**DOI:** 10.3390/s25195954

**Published:** 2025-09-24

**Authors:** Pablo Escapa Gordón, Vicente Matellán Olivera, Adriana Suárez Corona

**Affiliations:** 1 Department of Mechanical, Computer and Aerospace Engineering, Universidad de León, Campus de Vegazana S/N, 24007 León, Spain; vicente.matellan@unileon.es; 2 Department of Mathematics, Universidad de León, Campus de Vegazana S/N, 24007 León, Spain; asuac@unileon.es

**Keywords:** cybersecurity, V2V, V2X, vehicle communication, identity-based cryptography

## Abstract

Establishing secure vehicular communication is essential for the development of autonomous driving capabilities and to make new functionalities possible, such as smart traffic management, or systems aimed at avoiding or mitigating traffic accidents. In this scenario, where the deployment of a Public Key Infrastructure (PKI) may be difficult, the use of identity-based cryptography is proposed as a good alternative, because this approach simplifies encryption by enabling dynamic key generation and secure communication, without requiring prior key exchanges, making it highly scalable and efficient. In this way, this paper proposes a communication protocol applicable to V2V (vehicle-to-vehicle) and V2X (vehicle-to-all) communications, replacing schemes based on PKI with identity-based cryptographic schemes using the VIN (Vehicle Identification Number) as an unequivocal vehicle identifier. This paper also describes a prototype implementation in a conventional vehicle and the performance metrics of the system. Through a defined proof of concept, we obtained various quantitative results, demonstrating the importance of the processors used for the encryption and decryption operations required. The proposed system provides secure and flexible vehicle identification, with multiple practical applications. It can enables digital authentication, support toll payments without extra hardware, and facilitate V2X communication via the VIN for improved traffic management and safety. Additionally, it can streamline processes in repair workshops and optimize fuel payment and tracking at service stations.

## 1. Introduction

Modern-day vehicles are no longer composed of just hydraulic and mechanical components; they are increasingly becoming closer to robots (“computers with wheels”). Technologies such as Advanced Driver Assistance Systems (ADASs) [1] and external connectivity improve driver and passenger experience, and can also enable autonomous driving and smart traffic management. In this sense, one can consider cars as hyper-sensorized and connected platforms, with three goals for people transportation: no accidents, no pollution, and no traffic jams.

To realize these goals, three core elements—connectivity, safety, and security—are indispensable for the development and deployment of autonomous vehicles. In particular, vehicular communication entails a major revolution, since information exchange among vehicles (V2V communications), with infrastructure (V2I) (vehicle to infrastructure), or in general with other agents in traffic (V2X), could help to prevent the 1.35 million deaths caused by car accidents worldwide [2] and reduce the nearly 7 million deaths caused by pollution [3].

However, the car industry needs to guarantee the security of these communications to prevent their misuse. Misidentifying vehicles can lead to faulty driving decisions, resulting in avoidable safety risks and potential accidents. The aim of this paper was to design and evaluate a secure communication protocol for vehicular networks that replaces traditional PKI-based schemes with identity-based cryptographic mechanisms, using the Vehicle Identification Number (VIN) as a unique identifier. The main contributions of this research to the body of knowledge on V2V communication include a generic IB communication protocol, the use of the VIN as a secure and practical identifier for dynamic key generation in vehicular networks, a real-world prototype implementation demonstrating the feasibility of the proposed approach in a conventional vehicle, and an analysis of performance metrics that highlights the impact of hardware processing capabilities on encryption and decryption operations. In vehicular communications, we can highlight the following standards: IEEE 802.11p [4], ETSI TS 102 637 [5], ETSI EN 302 665 [6], ISO 21217:2014 [7], IEEE 1609.2 [8], IEEE 1609.3 [9], SAE J2735 [10], ISO 15118 [11], 5G V2X [12], ITU-T Y.2060 [13], and ISO 26262 [14]. All of these standards show us how V2X communications should function, offering different technical solutions. In these standards and other proposals, various methods are used, such as identification via IP addresses, ICCID (Integrated Circuit Card Identifier), random numbers, license plate readings [15], etc. In contrast, our contribution allows vehicles to be identified by their VIN, which is the simplest and most natural form of identification. The use of cryptography in these communication protocols enables authentication, which generates trust, enables confidential communication, and prevents the exchanged information from being modified. Some traditional V2X security protocols proposed decentralizing the Certificate Authority’s (CA) tasks to reduce the communication and computation overhead, as well as the dependence on a centralized CA in PKI-based solutions.

Identity-based cryptography, introduced by Shamir [16], is an alternative to public key cryptography that avoids the use of certificates and the management and updating of Certificate Revocation Lists (CRLs). In this case, arbitrary strings, called identities, can play the role of public keys. These must unequivocally identify users, such as email addresses or social security numbers in the personal communication setting. In the vehicular communication context, the VIN, which uniquely identifies vehicles, serves as an ideal candidate for such identifiers.

In this scenario, anyone can derive the public key of a user from his identity, and his private key is computed by a Key Generation Center (KGC) from the user’s identity and the KGC’s master secret key. In the context of vehicles, this process could be performed by the international normalization bodies in charge of issuing the World Manufacturer Identifiers (WMIs) of traditional frame numbers.

Moreover, when compared to credential management systems, identity-based cryptography avoids handling and securely storing multiple credentials, since key generation is inherently tied to the identities from which the keys are derived. This makes cryptographic schemes easier to integrate and improves scalability for systems with a large number of users, as in the case of vehicles. Privacy-friendly credential management systems also usually involve multiple steps for exchanging, verifying, and validating credentials, which can introduce delays or complexities in the authentication process.

Several identity-based cryptosystems have been proposed, including encryption, digital signature, and key establishment protocols. One can highlight the schemes by A. Shamir [16], D.Boneh and Franklin [17], C. Gentry and A. Silverberg [18], R. Sakai, K. Ohgishi and M. Kasahara [19], and C. Boyd and K. R. Choo [20]. Most of these make use of pairings (bilinear maps) [21], while some avoid their use to obtain more efficient protocols [22] or so that they can resist quantum attacks [23]. For our real test, we selected D. Fiore et al.’s [22] protocol, which can be applied over any cyclic group of prime order, and for which the Diffie–Hellman problem is intractable (no known efficient algorithm can solve it). This protocol is extremely efficient and only requires one round of communication with short messages, which makes it suitable for this application.

There are several applications for the proposed communication protocol called “V2V Secure communication protocol based on digital VIN number” and its generalization to V2X communications, such as the following:

- Digital documentation: This could allow us to authenticate a vehicle with law enforcement authorities. With a slight modification, new functionalities could be added, such as recording vehicle history, including data related to the vehicle’s repairs, owners, and technical reports.
- Tolls: The car could communicate with infrastructure without requiring any more specific hardware, making payments easier. This would be a universal system for identifying the vehicle and communicating the necessary information independently of the highway controller (the payment process is outside of the scope of this paper).
- Payment method: Unequivocal identification can allow vehicles to serve as payment gateways, making applications possible in places such as gas stations, fast food kiosks, etc. The integrated On Board Unit (OBU) could have a graphic console, adapted to the navigator screen, so that these kinds of transactions could be accepted.
- V2X Identification: The information coded in the VIN provides details about a vehicle’s features. Therefore, its identification against other components is very useful for information exchange. This has multiple applications: determining the length of a vehicle, locating a pedestrian, creating digital signage, providing traffic jam solutions, etc.
- Repair workshops: Currently, the reception process at repair workshops is often tedious because a lot of time is required to make a repair order. This period could be shortened with our communication system, since cars would be able to very quickly send the owners’ details and information about failures or servicing to the workshop’s admission area.
- Gas stations: The system could avoid accidental fuel refills and allow a much faster payment.

The manuscript is organized in the following way: Section 2 presents a review of the state of the art in secure V2V communication protocols, highlighting the most relevant approaches and their limitations. Section 3 describes the proposed protocol in detail, specifically designed to ensure secure communication between vehicles using identity-based cryptography. Section 4 and Section 5 present the experimental validation, evaluating the feasibility and effectiveness of the protocol. Section 6 summarizes the main findings of the work, emphasizing the practical utility of the developed protocol. Finally, Section 7 discusses potential future research directions aimed at improving and extending the proposed system.

## 2. Related Work

As with any other type of computer system, being able to identify users is essential to develop applications that require authentication systems. In this sense, V2X applications are no exception.

Vehicular communication can be classified into two types: internal, which includes transmissions among different Electronic Control Units (ECUs), usually based on the CAN Bus (Controller Area Network) protocol [24], and external, known as V2X (vehicle-to-everything) [25], which links vehicles to the environment through radio frequencies, WiFi, or 5G connections and which is mainly defined by the IEEE 802.11P [26] and 5G [27] standards. V2X communications are usually classified as follows [28]:

- V2V: connections among vehicles, in order to, for example, avoid collisions; vehicles can share position and speed data to interact and be able to react faster than the driver.
- V2P (vehicle-to-pedestrian): connections between vehicles, pedestrians, or riders for detection, alert notification, and accident prevention.
- V2I: connections between vehicles and road infrastructures to manage the priority and timing of traffic signs, optimizing the traffic.
- V2N (Vehicle-to-network): connections between vehicles and the cloud to route traffic, to develop real-time traffic management, Beyond the Visual Line Of Sight (BVLOS) warnings, etc.
- V2H (Vehicle-to-home): connections between vehicles and homes to implement, for example, door opening once the car is parked.

Security and privacy issues in vehicular networks have been extensively reported in the literature. Secure communication is essential for the adoption of V2X technologies, since it is required to ensure the safety and security of future connected and autonomous cars.

To carry out a complete assessment of the state of the art in V2X communications, we have organized this section into an analysis of technologies for the secure identification of vehicles, an analysis of communication standards (mainly based on WiFi and 5G), and an examination of V2V secure communication protocols.

### 2.1. Vehicle Identification

Vehicle identification is an essential phase in ITSs (Intelligent Traffic Systems), and so there have been many studies on the topic. Some of them rely on car license plate recognition, which was developed in 1976 at the Police Scientific Development Branch (PSDB) in the UK [29].

Johnson and Bird [30] proposed knowledge-guided boundary following and template matching for automatic vehicle identification. In 1990, Lotufo, Morgan, and Johnson [31] proposed automatic number plate recognition with optical character recognition techniques. Later, Fahmy [32] proposed Bidirectional Associative Memory (BAM) neural networks for number plate reading. In 2010, Changshui et al. [33] showed how an identification system could be used to analyze vehicle handling and stability, and subsequently, Jeevagan et al. [34] proposed the development of vehicle identification using Radio Frequency Identification (RFID).

Later, in 2018, Satsangi et al. [15] compared thresholding optical character recognition (OCR) and machine learning approaches for license plate recognition, and in 2019 [35], Kohli and Sharma studied the possibility of identifying vehicles through GSM (Global System for Mobile Communications) signals.

The use of the VIN and identity-based cryptography in car-related communication has already been proposed by Groza et al. in 2020 [36], but our work goes one step further by using this concept in a digital version.

In 2023, ref. [37] proposed a solution involving density-based traffic management using Arduino, a GSM module, and RFID. Also in 2023, ref. [38] proposed a cooperative method to identify vehicles in autonomous driving using registration numbers (VRNs). Currently, blockchain technology is also being used for vehicle identification. For instance, the proposal in [39] illustrated how its use facilitates identification through the various physical forms of vehicles. The issue of vehicle identification was also present in [40], who explained how to create an efficient authenticated key agreement scheme, titled STCLA, designed for fog-based IoV, adopting certificateless cryptography. It is also worth highlighting that the latest studies on vehicle recognition using license plates with the Yolo V8 algorithm achieved an overall recognition accuracy of 98.05 percent [41]. Our study shows that the identification of vehicles can be achieved in different ways, including identification through their license plate, through an RFID device, or even through their form, but no article has yet shown the possibility of identifying vehicles by the chassis number in digital format, in Table 1 we can see a classification.

### 2.2. WiFi vs. 5G Communications

After having studied vehicular connections, we can describe them as emerging networks whose principal technologies are WiFi and 5G. In this section, we will review their main characteristics, together with their advantages and disadvantages.

**WiFi** under the 802.11P standard, also known as WAVE (Wireless Access for the Vehicular Environment), has the key features shown in Table 2. It represents a positive development with respect to the IEEE 802.11A standard, since it has physical and MAC (Media Access Control) improvements. Their importance lies in the fact that they serve as the basis for direct V2V communications.

This kind of communication is known as DSRC (Dedicated Short Range Communication) and is ideal for providing real-time support to cooperative systems [42].

**5G** or Cellular C-V2X is a communication technology with a shorter range that employs current mobile phone coverage using license specter networks. It connects large spaces and can be used for V2I services that require less latency (Technology 3G and 4G LTE Release 14). The new 5G (3GPP Release 16) will be the key enabler for V2X, with three main features: ubiquitous connectivity for large sets of users, very low latency, and high-speed Gigabit connection. Its principal characteristics are presented in Table 3.

The WiFi 802.11P DSRC and 5G C-V2X standards are supported by large committees. The best way to increase road safety and decrease road fatalities is not to choose one standard, but to use a hybrid communication approach in which WiFi [5.9 GHz] and 5G [3.x GHz] technologies work together.

### 2.3. V2V Secure Communication Protocols Using Identity-Based Cryptography

Nowadays, countless applications, papers, patents, and research studies exist related to vehicular communications, but none of them have used the VIN in digital format as a car identifier. We will summarize the main results in this field.

V2V communication is an emerging technology that helps nearby moving vehicles communicate with each other. In this way, Vibin et al. [42] presented the design and development of a V2V communication system that collects vehicle-related data and transmits it over WiFi to the vehicles in its vicinity.

New car equipment applications like longitudinal and lateral vehicle following control systems that use radar can help to reduce traffic jams. For instance, Wei et al. [43] proposed a method to calculate the trajectory of the preceding vehicle based on its historical movement data transmitted among vehicles.

Car manufacturers, insurance companies, workshops, and telecommunications operators, etc., are aware that, in the near future, new connectivity services will create new models of business. A good example is the car manufacturing company SEAT, which has created a strategy for urban mobility called SEAT CODE [44].

Zhao et al. [45] presented a system to create car platooning by adjusting their distance according to traffic using an ECAN module to communicate with the CAN bus. Hwang et al. [46] described a new method that responds with the necessary ECUs for a specific V2V message. None of these developments were carried out from the perspective of cybersecurity. Identity-based (IB) cryptography has previously been used for V2V communications [47]. Andreica and Groza [48] identified vehicles and used this identification number to bootstrap security based on identity-based cryptographic schemes. They also introduced the use of license plates as a means to identify vehicles by applying identity-based schemes [48]. In the VANETs (Vehicle Ad hoc Networks) setting [47], Shuhaimi and Juhana [47] studied IB schemes in order to provide security and privacy for VANETs. In other situations where privacy is required, other proposals using pseudonyms (random identifiers) [49] or group signatures have been used [50]. In 2025 N. Gopinath, K. L. Narayanan, S. Sageengrana, and M. Meenaksh studied a hybrid authentication architecture for VANETs combining certificate-based and identity-based cryptography with machine-learning-based attack detection, achieving significant improvements in latency, scalability, and detection accuracy [51]. However, most of these proposals did not specify which information is used for the identity.

In some proposals, the authors specified the identity used. For example, in [48], license plates were used as identities. They are first recognized by smartphone cameras located in the vehicles. However, the string that is usually used for vehicle identification is the Vehicle Identification Number, since it is unique, while the license plate number can change if the vehicle is reregistered, for example, in a different country. This identity was used by Groza et al. [36] for car access control with a smartphone. However, they recognized the VIN using a smartphone and did not use its digital version.

## 3. Identity-Based V2V Secure Communication Protocol

As previously discussed, it is necessary to design and implement a secure communication protocol, where communications can be performed in a limited time. Latency and computing capacity restrictions make traditional approaches ineffective; thus, we propose a protocol that uses the identities of vehicles.

In this section, we propose a generic communication protocol between two vehicles which guarantees confidentiality, authentication, and integrity. This protocol builds on a one-round two-party Identity-Based Key Establishment Scheme and an AEAD (authenticated encryption with associated data) scheme [52], and uses the Digital Vehicle Identification Number as the identity. The protocol begins with the establishment of a session key using a one-round key exchange scheme based on identity-based authentication, followed by an AEAD scheme for message exchange between the vehicles.

There is a preliminary setup phase where the system’s public parameters and the private key of the KGC are established. The authority responsible for this management calculates each vehicle’s private key, based on its VIN and secret information, and provide this to the vehicle.

Subsequently, message exchange between the two vehicles occurs, with authentication based on their digital VINs. Vehicle B adds a Message Authentication Code (MAC) to the message sent, calculated using the shared key generated from the message sent by vehicle A. The exchange concludes with vehicle A sending an MAC, calculated using all exchanged messages, to vehicle B as a key confirmation. Once the vehicles have shared the common key, they can begin exchanging information using an authenticated encryption scheme.

### 3.1. The Digital Vehicle Identification Number

The VIN is the usual way to uniquely identify a vehicle for administrative purposes. It is equivalent to a passport or national ID numbers for people.

**Figure 1:**
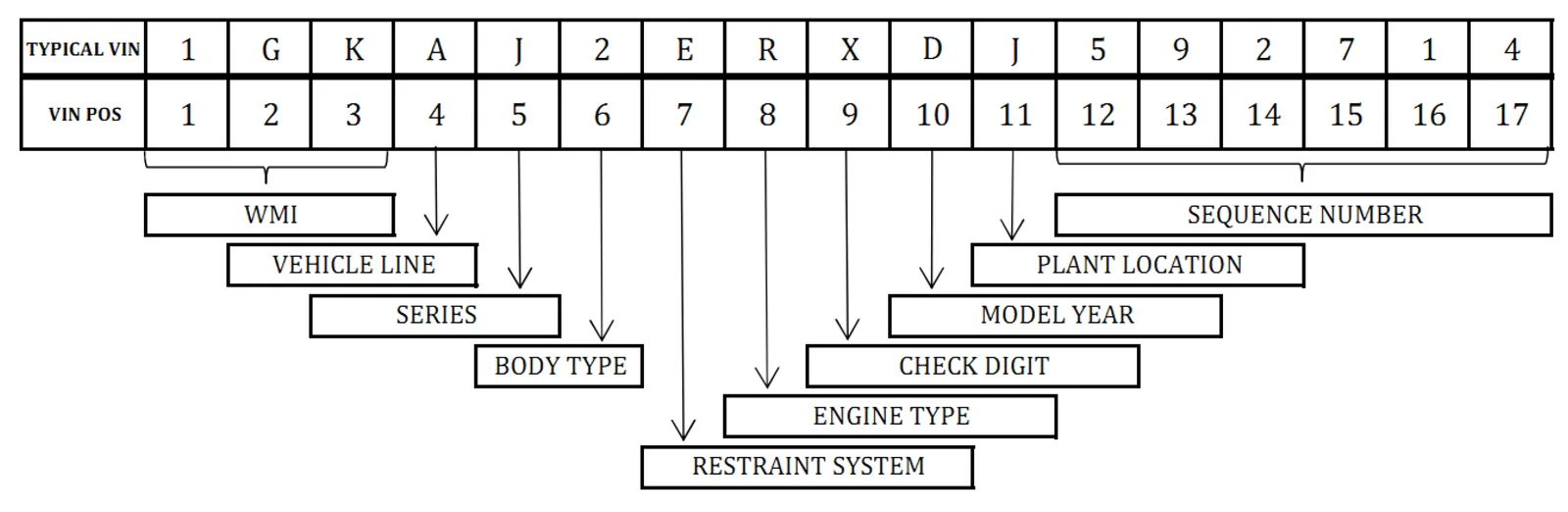
VIN decoding [54].

In its current format, the VIN comprises 17 characters and was standardized in 1983 by ISO 3779 [53], which defines its structure as showed in Figure 1. There are some differences between the European and American formats, and the characters can be either numbers or letters (except for I, O, Q, and Ñ). The VIN has to be easily readable and located in a safe, tamper-proof place. It is usually located under the hood or inside the vehicle, directly engraved in the frame.

Although there are many options for identifying vehicles, the main approach is obtaining the VIN directly from the car’s documents or from the vehicle frame; the problem with this method is that it is very slow and not a digital solution. It is employed, for instance, in workshops and technical inspections. Another way to achieve this is by reading the number plate, with the problematic aspect of relying on number plates, which can be changed or altered. This is used in public parking areas and by traffic control cameras. Moreover, RFID systems can automate identification when vehicles pass through tolls, but they cannot identify the car, they only identity an RFID system. Recent work has proposed a CNN-based approach combining data augmentation and multi-task learning for efficient and accurate vehicle model identification in forensic contexts, improving upon traditional manual methods. [55].

A digital version of the VIN has recently been proposed [56]. In this patent claim, this digital VIN can be accessed through the OBD2 (On-Board Diagnostics 2) diagnosis socket, mandatorily present in vehicles under the SAE J1979 standard [57] and its European counterpart, the ISO 15031 [58].

The OBD2 standard can provide us with this number using a simple and universal instruction PID 0x02 issued to any vehicle. This instruction returns the VIN extracted from the ECU in hex format.

### 3.2. Cryptographic Tools

To build our V2V secure communication protocol, we make use of different cryptographic tools, both symmetric and asymmetric. In this section, we describe their main features regarding security.

#### 3.2.1. Message Authentication Codes

Integrity and authentication are essential cryptographic goals. To achieve them with symmetric cryptography tools, one can make use of *Message Authentication Codes (MACs)*.

To achieve this, when party A sends a message to party B, A appends to the message an authentication tag, computed by the MAC algorithm as a function of the message and the shared secret key. Party B, when receiving this information, recomputes the authentication tag in the received message using the MAC with the shared key and checks whether the obtained value is equal to the received tag. In this case, one can see that the message has not been altered and has been sent by a party knowing the shared secret key.

Some efficient constructions of message authentication schemes are based on cryptographic hash functions and derive their security from these. We use the HMAC (Hash-based Message Authentication Code) construction, with the SHA-256 hash function [59].

#### 3.2.2. Authenticated Encryption with Associated Data

AEAD is a symmetric tool that provides both confidentiality and authentication for the encrypted text, and also allows us to check the integrity and authentication of additional associated data that are sent in the clear. It was introduced by Rogaway [60].

It can be built by combining MACs and symmetric ciphers, using block cipher modes of operation. There are dedicated algorithms for this task.

In our proof of concept, we use AES-256 (Advanced Encryption Standard) with the Galois Counter Mode (AEAD_AES_256_GCM), because AES provides faster performance, lower resource usage, and high efficiency.

#### 3.2.3. Identity-Based Key Establishment Scheme

Key establishment schemes allow two or more users to agree on a common key that can afterwards be used in a symmetric key algorithm. These protocols are usually interactive, and each round of messages that are sent at the same time is called a round.

A desired security requirement is that the protocol is authenticated; i.e., the users are sure about who they are agreeing the key with. For this, a Public Key Infrastructure might be needed, with its corresponding shortcomings.

To avoid such disadvantages, an identity-based approach can be used, where users only need to know their counterpart’s identity to establish a key in an authenticated manner. The KGC is in charge of creating the private keys corresponding to the identities and sending them to the users.

An instance of the protocol is called a session, which has different variables associated with it, indicating, for example, the session state, the peers, whether the session has aborted, the session identifier, etc.

The adversary controls all the communication and can insert, delay, modify, or intercept the messages exchanged. Moreover, it has access to different information, such as the private keys of users, session keys, or the ephemeral information of the session. A party is said to be *corrupted* if the adversary has obtained its private key, all its session states, and all session keys currently stored, and is controlled by the adversary.

**Definition** **1.** *An identity-based key-agreement protocol is said to be secure if, for any probabilistic polynomial time adversary A, the following holds:*

- *If two parties not corrupted by the adversary complete matching sessions, then they will compute the same session key with overwhelming probability;*
- *The probability that A guesses the correct bit b when it is provided either with a random session key (if the randomly chosen bit is 1) or with the real session key (if the randomly chosen bit is 0) is at most $\frac{1}{2}$ plus a negligible function on the security parameter.*

We refer to [22] for a more formal description of the security model.

In our proof of concept, we use the protocol of Fiore et al. [22]. A description can be found in Appendix A.

### 3.3. Secure V2V Communication Protocol

In this section, we propose a secure communication protocol. It builds on a one-round two-party Identity-Based Key Establishment Scheme, so that two vehicles can agree on a key to be used afterwards with an AEAD scheme [52].

**Figure 2:**
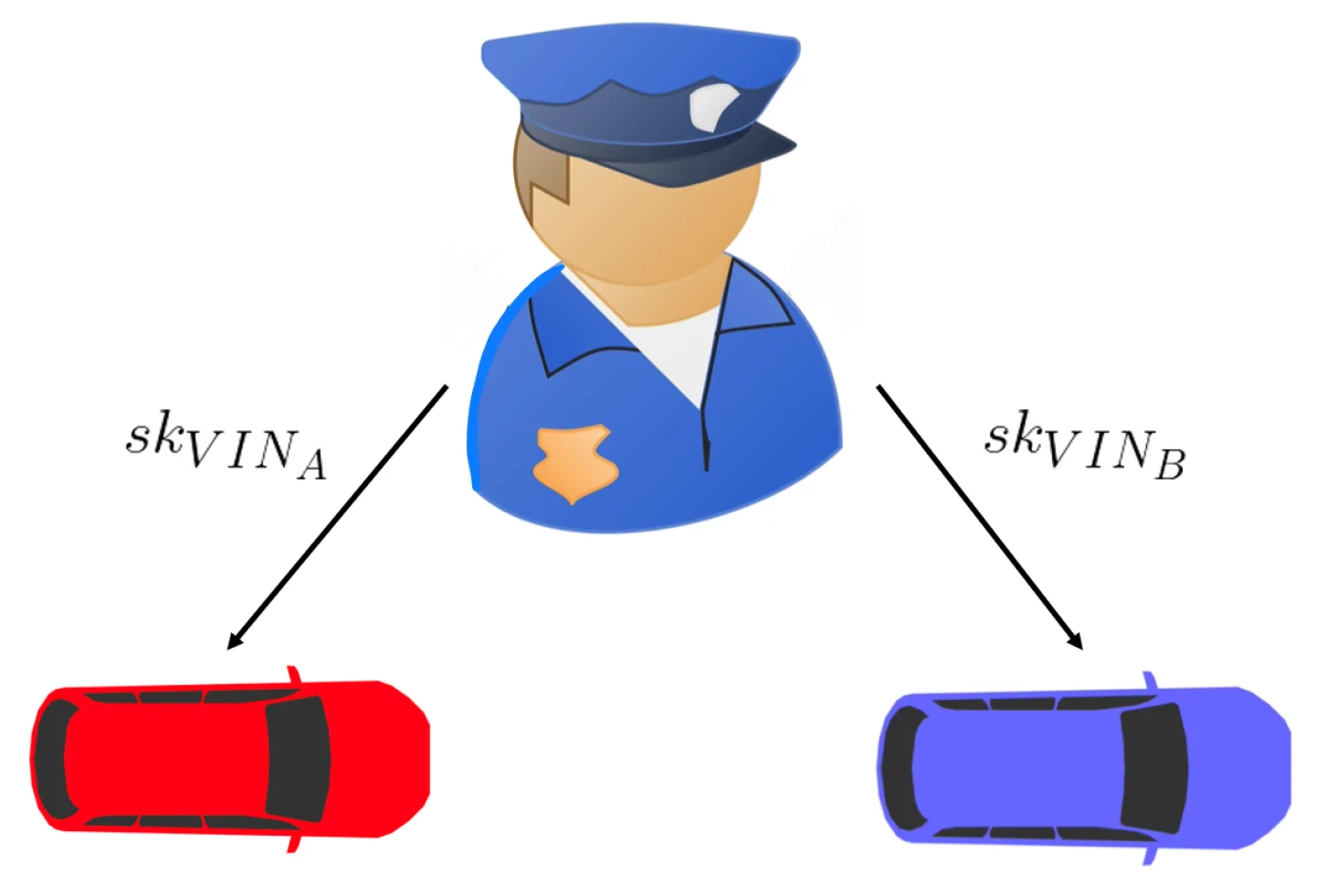
Generic V2V communication protocol. Vehicle private keys.

Before the exchange of messages, there is a setup phase, where public parameters and the master secret key of the Key Generation Center (KGC) are established. The KGC, to create a private key for a vehicle, takes its $VIN_{veh}$ and the master secret key to compute $SK_{VIN_{veh}}$. The KGC sends each vehicle its private key, as shown in Figure 2.

**Figure 3:**
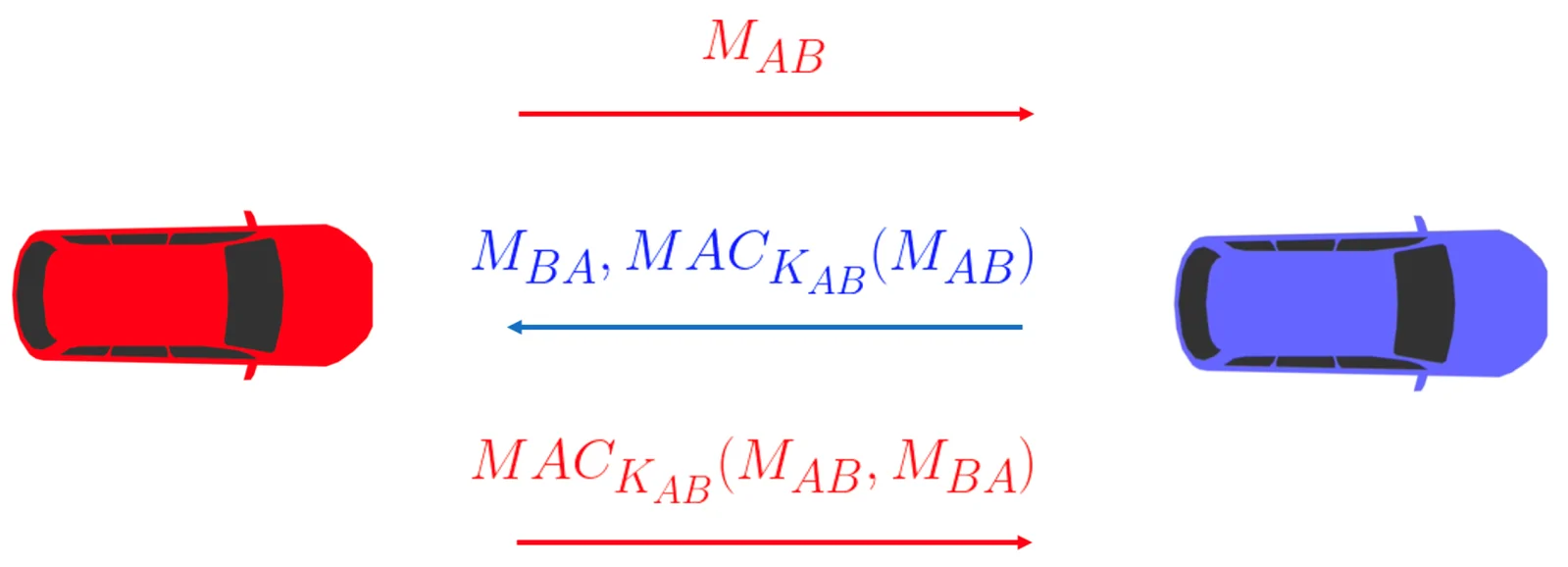
Generic V2V communication protocol. Communication flow.

Afterwards, both vehicles exchange messages, authenticated based on the digital VIN, and moreover, they compute the tag generated by the MAC in the message sent by vehicle *B* and the common key established with vehicle *A*. The communication ends with vehicle *A* sending a tag from all the messages exchanged with the common key established, acting as key confirmation. The protocol is sketched in Figure 3.

Once the vehicles have shared a common key, they use an AEAD scheme to securely communicate.

This protocol is secure for any secure one-round two-party Identity-Based Key Establishment Scheme.

**Theorem** **1.** *Let P be a one-round two-party Identity-Based Key Establishment Scheme which is secure in the sense of Definition 1; then, the proposed communication protocol provides confidentiality, integrity, and authentication.*

In our proof-of-concept implementation, to instantiate the proposed protocol, we used the IB key establishment scheme by Fiore and Gennaro [22], which is secure under the *Strong Diffie–Hellman Assumption*. A description of the protocol can be found in Appendix A, and we used HMAC-SHA256.

**Figure 4:**
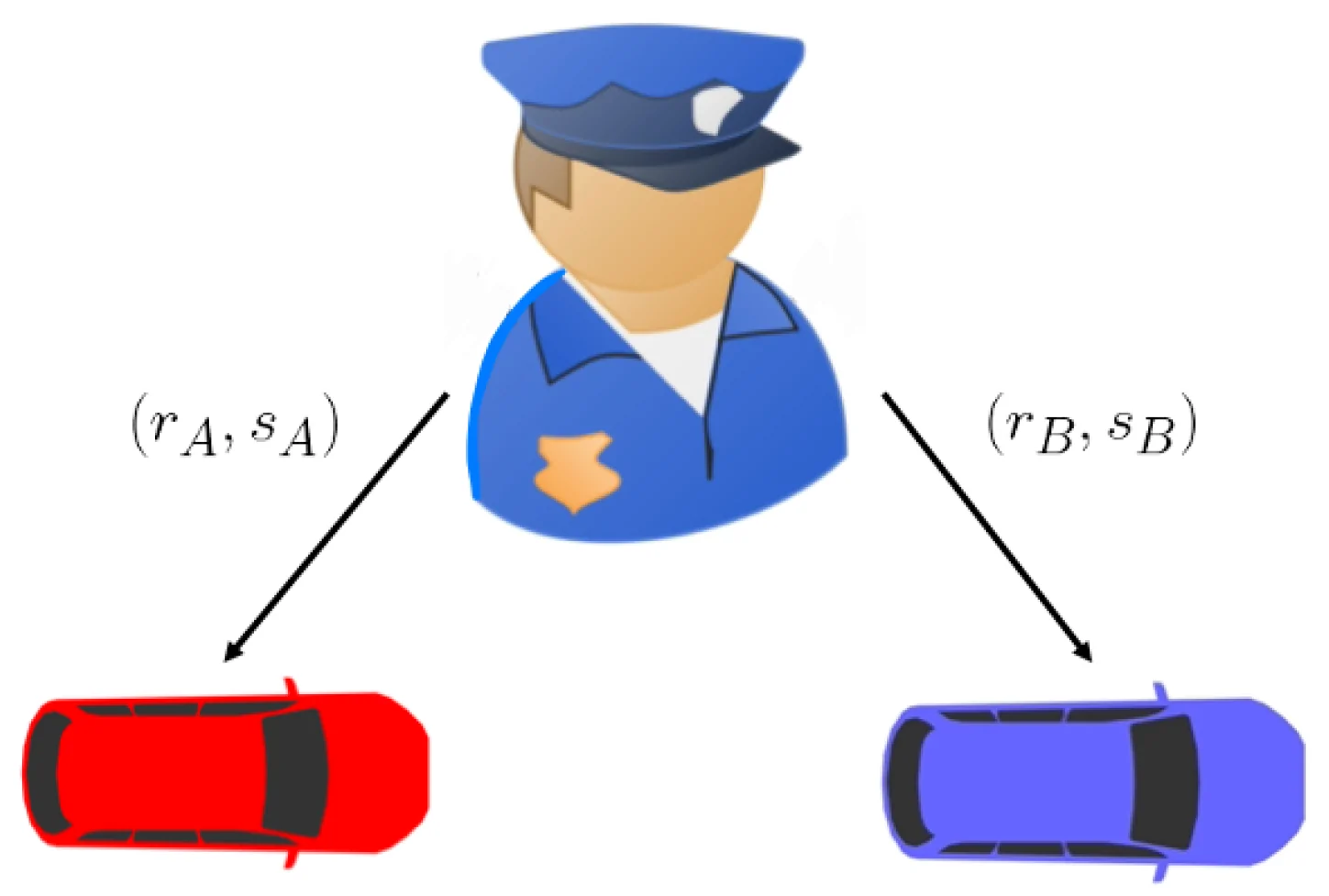
V2V communication protocol based on [22]. Vehicle private keys.

The proposed protocol inherits the security from Fiore and Gennaro’s construction, and therefore, its security relies on the strong Diffie–Hellman assumption in the Random Oracle Model (see [22] for the security proof). This protocol is very efficient, since it does not make use of bilinear maps, which makes it suitable for this scenario. The vehicle private keys generated by the KGC are shown in Figure 4.

**Figure 5:**
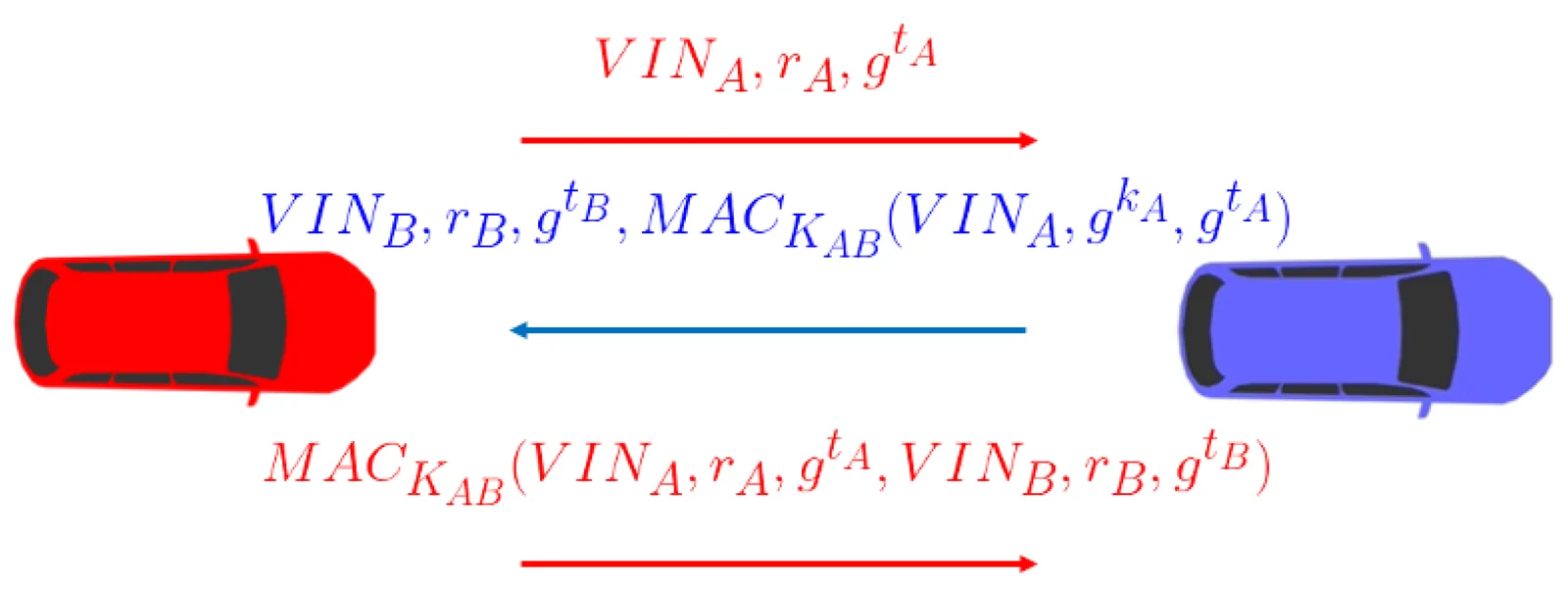
V2V communication protocol based on [22]. Communication flow.

The communication flow of the protocol instantiated with Fiore and Gennaro’s IB Key Establishment Scheme using the VIN as identity can be seen in Figure 5.

**Figure 6:**
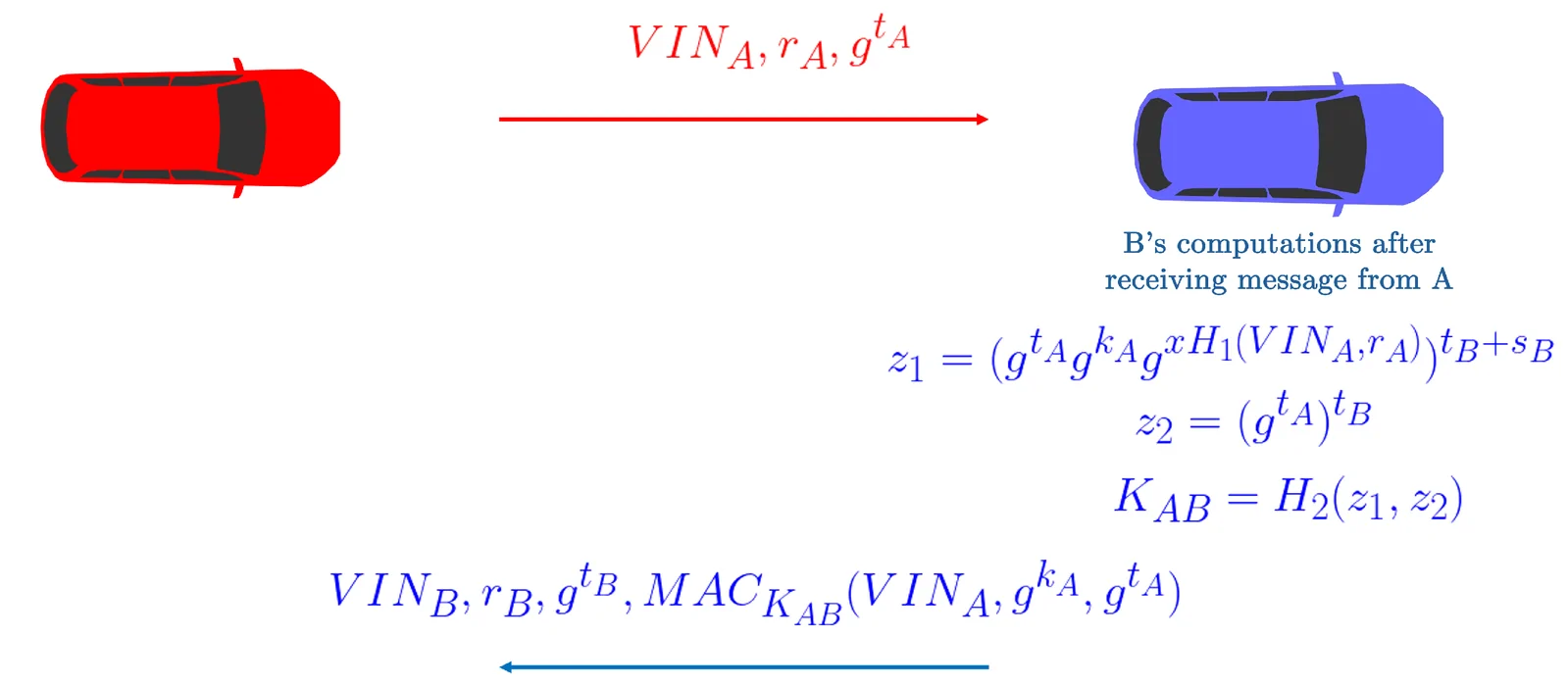
V2V communication protocol based on [22]. B’s computations.

**Figure 7:**
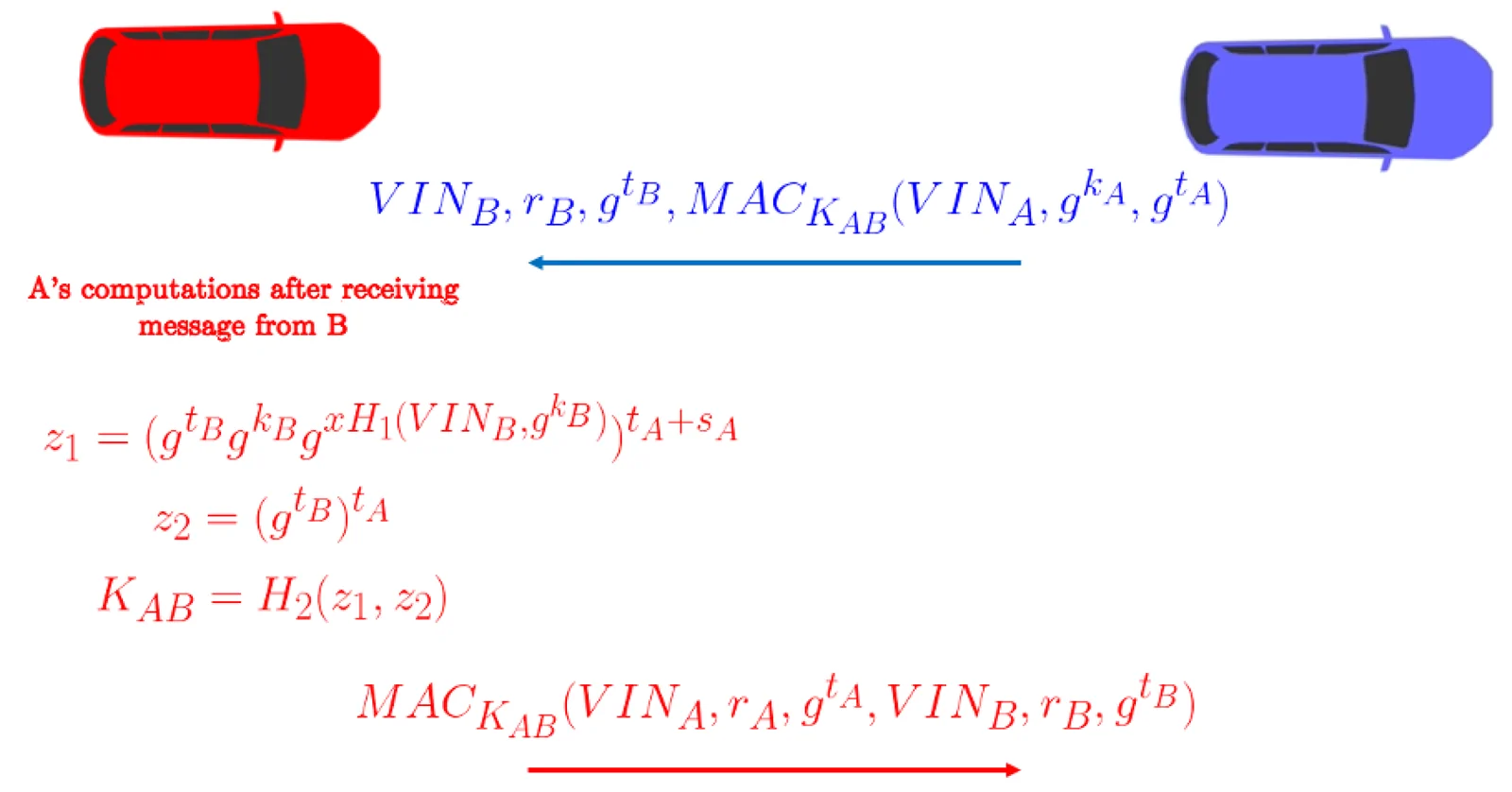
V2V communication protocol based on [22]. A’s computations.

Moreover, Figure 6 and Figure 7 show the intermediate computations performed by vehicles *B* and *A*, respectively, after receiving the other vehicle’s message.

Subsequently, we use the established key for an AEAD. In particular, we use AES-256 with the Galois Counter Mode (AEAD AES 256 GCM).

We decided to use AEAD since it combines both encryption and authentication in a single, efficient operation, which makes it a strong choice for secure communication and data protection.

In particular, we chose AES-256 with the Galois Counter Mode, since it provides faster performance, lower resource usage, high efficiency, and strong security. AES is the current standard for block ciphers and has been widely analyzed and used over many years. Moreover, AES-GCM is widely supported in both hardware and software implementations, making it a highly accessible choice. In addition, modern processors have built-in support for AES operations, which further boosts the speed of AES-GCM.

## 4. Proof of Concept

The general aims of the built PoC were as follows:

1. To demonstrate that the VINs of the two cars can be read through their own CAN Bus.
2. To validate that it is possible to create a secure WiFi communication between both cars, using the proposed protocol based on Identity-Based Cryptography, described in Section 3.3.
3. To measure the processing time needed to send a message or to establish a common key and exchange a message using an AEAD in two different scenarios.

To summarize, the PoC demonstrated that the digital VIN is a secure and viable way to identify vehicles and showed two different applications. It was very important to know the processing time, since many uses require a near-real-time communication system. We show that the solution proposed is viable and that we could characterize its main quantitative metrics, to measure and quantify its computational performance.

The analysis of the logs obtained in the PoC enabled us to define the specific hardware needed to comply with latency requirements. It was important to assess how vehicle speed would affect the proposed system.

A prototype was implemented and tested in a real scenario. The goal of the prototype was to show that data can be sent from the CAN bus of the first vehicle to the second vehicle. To ensure secure transmission, we needed to obtain VINs through the OBD2 diagnostic port to use them as identifiers.

The method was as follows: First, once the setting is configured (all elements are in the same WiFi network) and we have connected all the elements, the VINs of both cars are read. This can be accomplished by sending the PIDs (Parameter IDs), which are codes used to request data from a vehicle, used as a diagnostic tool. In Table A1, we can see the PIDs needed to obtain the VIN. Afterwards, we establish an encrypted connection using the VINs obtained by implementing the IB-based scheme described in Figure 6 and Figure 7. This protocol generates a common key that must then be used for the AEAD. Finally, speed or GPS details can be requested and sent between both cars.

## 5. Experimental  Validation

### 5.1. Experiments

**Figure 8:**
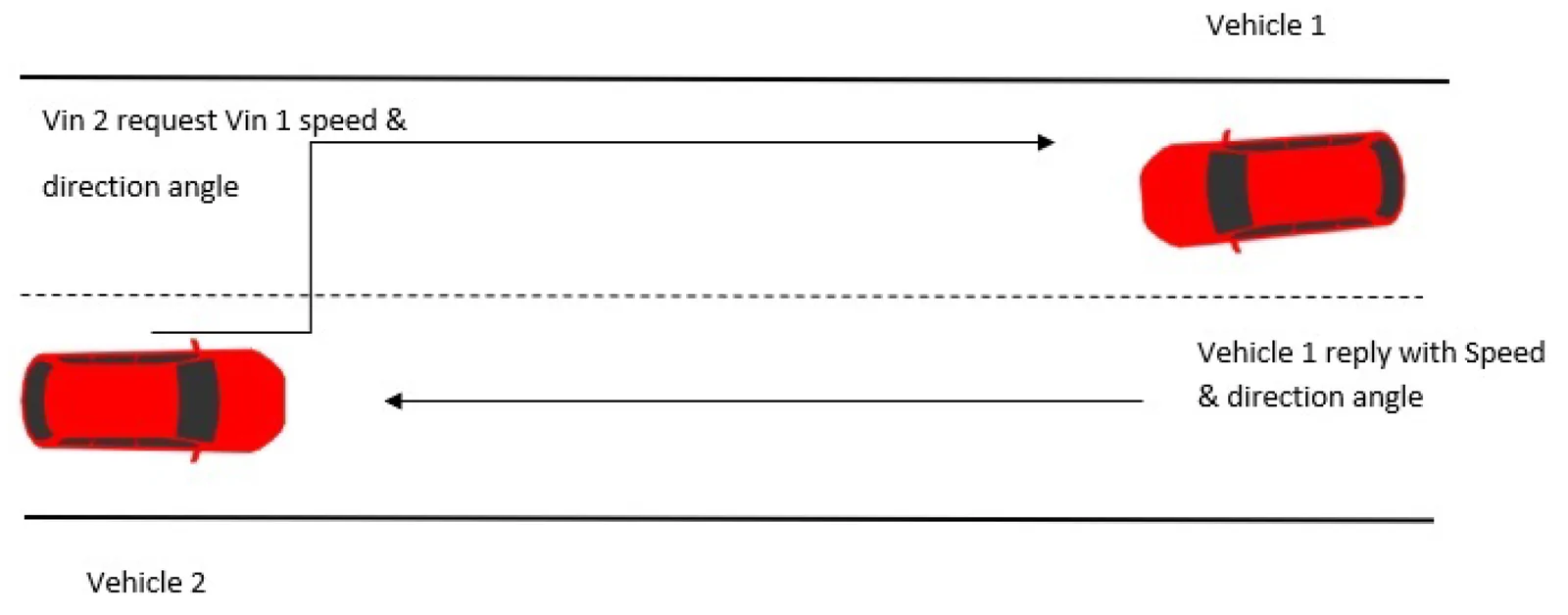
First experiment: a collision avoidance system.

**Figure 9:**
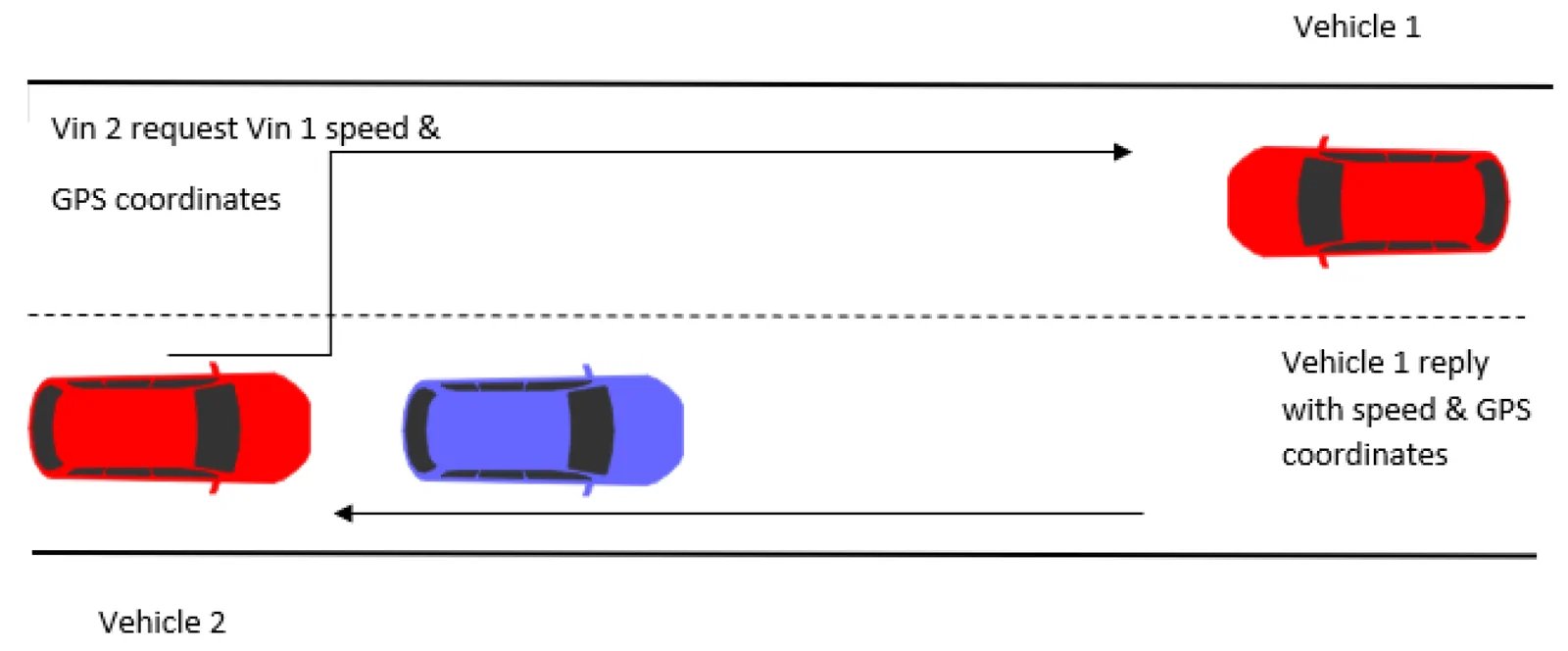
Second experiment: overtaking.

This section describes two different scenarios in which we implemented vehicular communication:

1. The first experiment, represented in Figure 8, demonstrated the ability to use the designed OBU to read one or several CAN bus parameters. In this case, we requested the speed and direction angle of vehicle 1, which moved in the opposite direction to vehicle 2. The processing of this information could be used to develop a collision avoidance system.
2. In the second experiment, described in Figure 9, vehicle 2 requested the position and speed of vehicle 1. This request was managed by the OBU, which had a GPS module that was able to alternatively obtain this information directly from the CAN bus (this is possible only if vehicle 1 has this option). The processing of these data will not supply enough information to know whether one vehicle can pass the other, but if these data are combined with the speed data and other details from the first vehicle like horsepower, speed gearbox, etc., one can determine the viability of this action.
  We tested the proposed protocol in both scenarios. In the first, both cars were stationary and there were five meters between them, and in the second, the first car was stationary and the second was moving at 5 km/h. This allowed us to measure the computation overhead when sending various messages (speed, temperature, etc.).

### 5.2. Materials

**Figure 10:**
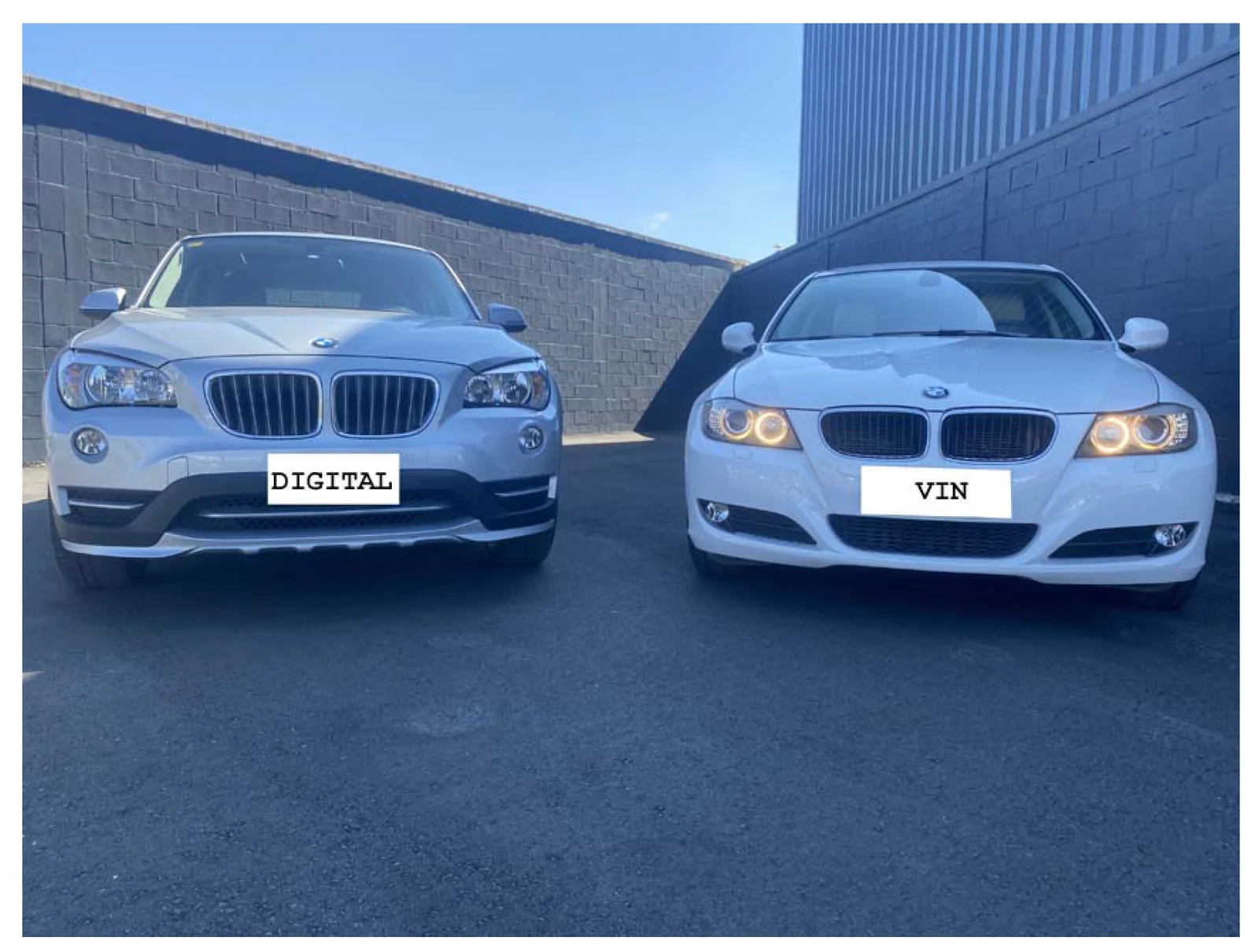
Vehicles used in the experiments.

The first vehicle showed in Figure 10 was a BMW X1 with VIN WBAVN71060VX14326 and equipped with a Raspberry Pi 3 and a PiCAN module, whose principal components are presented in Table 4. This set formed the OBU embedded in the first vehicle, equipping it with extra applications like encryption protocols and connectivity.

The second vehicle was equipped with an Asus laptop which connected the BMW 320D vehicle with VIN WBAUT31010F150820 through the CANalyze interface. Its main components are shown in Table 5.

**Figure 11:**
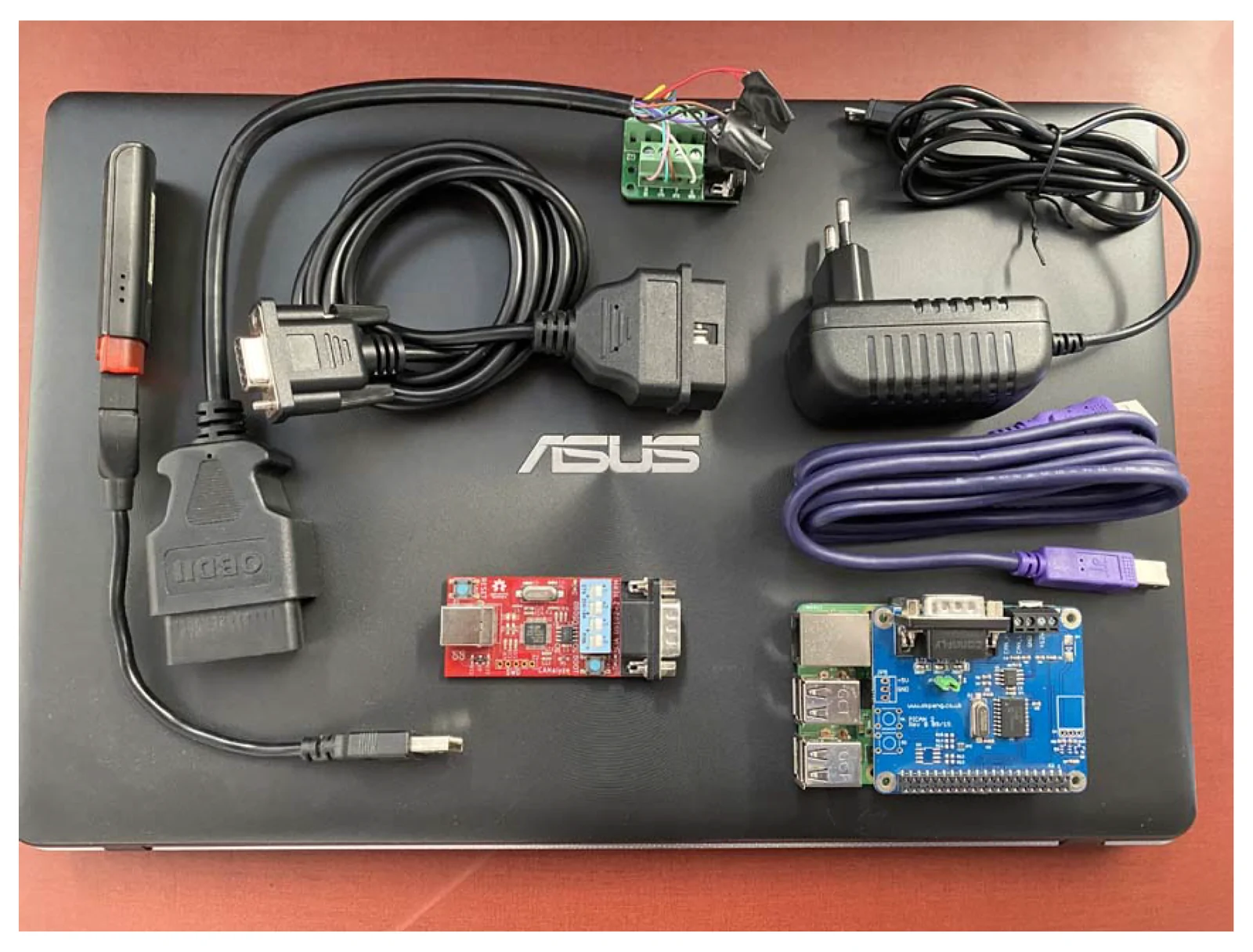
Hardware used in the experimental validation [54].

The final components for this proof of concept are presented in Table 6 and consisted of a WiFi network created by a TP-Link router, and the central server from which the laptop and the Raspberry could be controlled remotely. All hardware is shown in Figure 11.

### 5.3. Methods

Our method was based on iterating over ten samples to test the following variables: First, both cars can read the VIN through the OBD2 port. Second, they can establish communication. Third, they can exchange public keys. Fourth, they can send messages. Fifth, they can encrypt and decrypt messages. Sixth, they can perform the complete process; that is, they are, for example, capable of sending data such as speed between them in an encrypted message with integrity and identity-based authentication. We then established the computational performance they required. We used Sysbench with the Raspberry Pi and CPU-Z with the Asus notebook.

**Figure 12:**
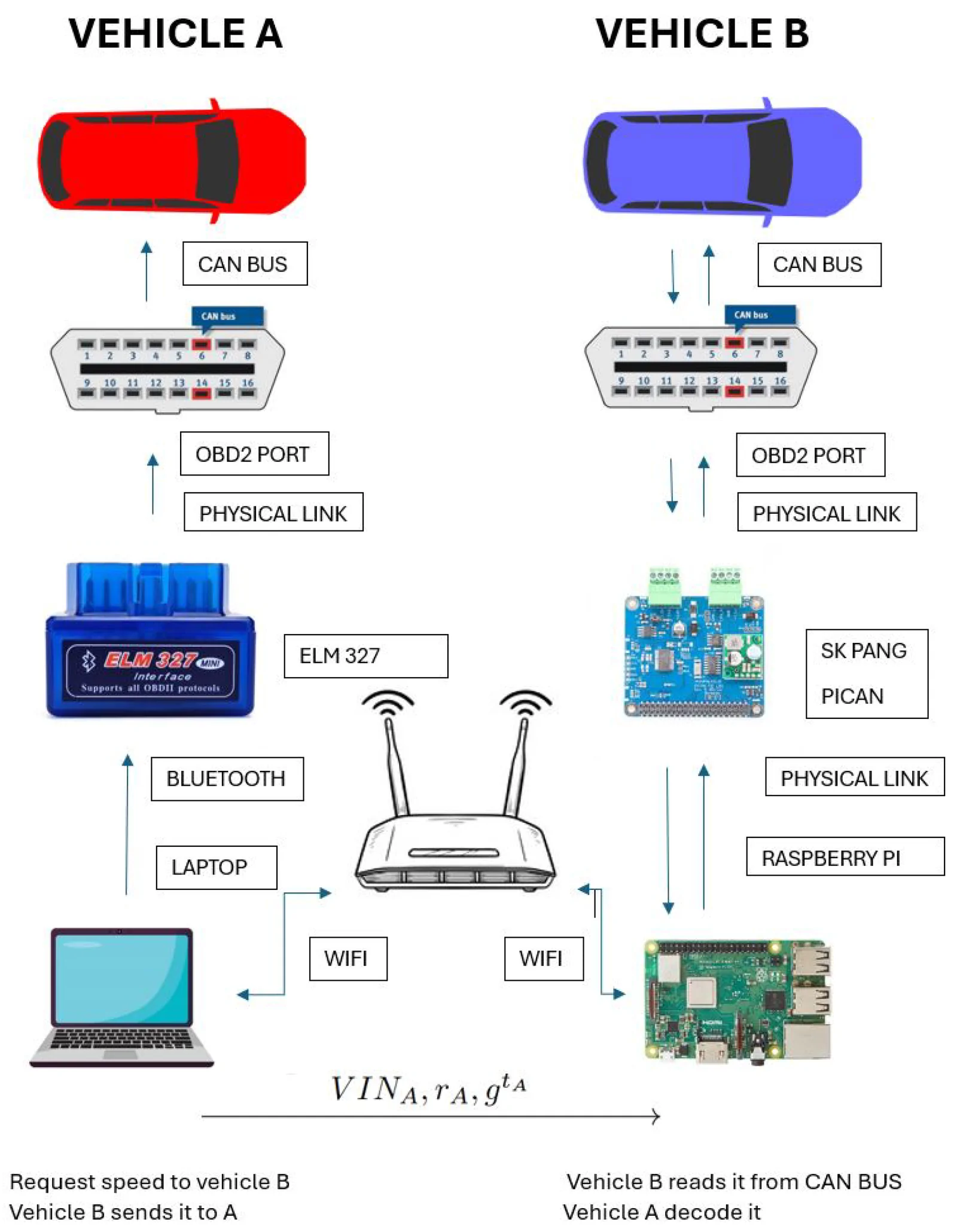
Schematics of the experiment.

Figure 12 shows the connections of all components in a schematic form.

For this proof of concept, we used the IEEE 802.11ac standard, which offered optimal results in establishing connections, except in terms of latency. We set up both devices as an ad hoc network, so that they could interact in a decentralized manner without needing a WiFi router [61]. This could be accomplished by activating Soft AP in the wireless network interfaces, to have direct WiFi through a peer-to-peer (P2P) WiFi. There are several studies where the deployment of V2V communication has been used, such as [62,63]. For each quantitative variable, we computed the average, variance, and standard deviation.

### 5.4. Experimental Results

Experiments were performed to test the proposed communication system and to measure the operating time of the system.

We applied the proposed Identity-Based Key Establishment Scheme. In Table 7 and Table 8, we can see its computational performance. As expected, the Asus laptop with a 3.2 GHz processor was much faster than the Raspberry Pi. We can also see that the vehicle speed affected the processing time. This delay was caused by the wave transport medium with the new 5G or 802.11p. However, this effect was minimal and could be adjusted to new latency requests. The test showed that the algorithm worked properly. Table 9 and Table 10 show the average, variance, standard deviation, and coefficient of variation of the measured metrics. The time obtained was the complete time needed to send information from the first vehicle to the second, such as speed. This included the time necessary for the cryptographic scheme, communication delay, and processing time in both vehicles.

The results show that the systems worked properly in the ten samples. The average performance was optimal for V2V applications, with very low standard deviation.

### 5.5. Discussion

**Figure 13:**
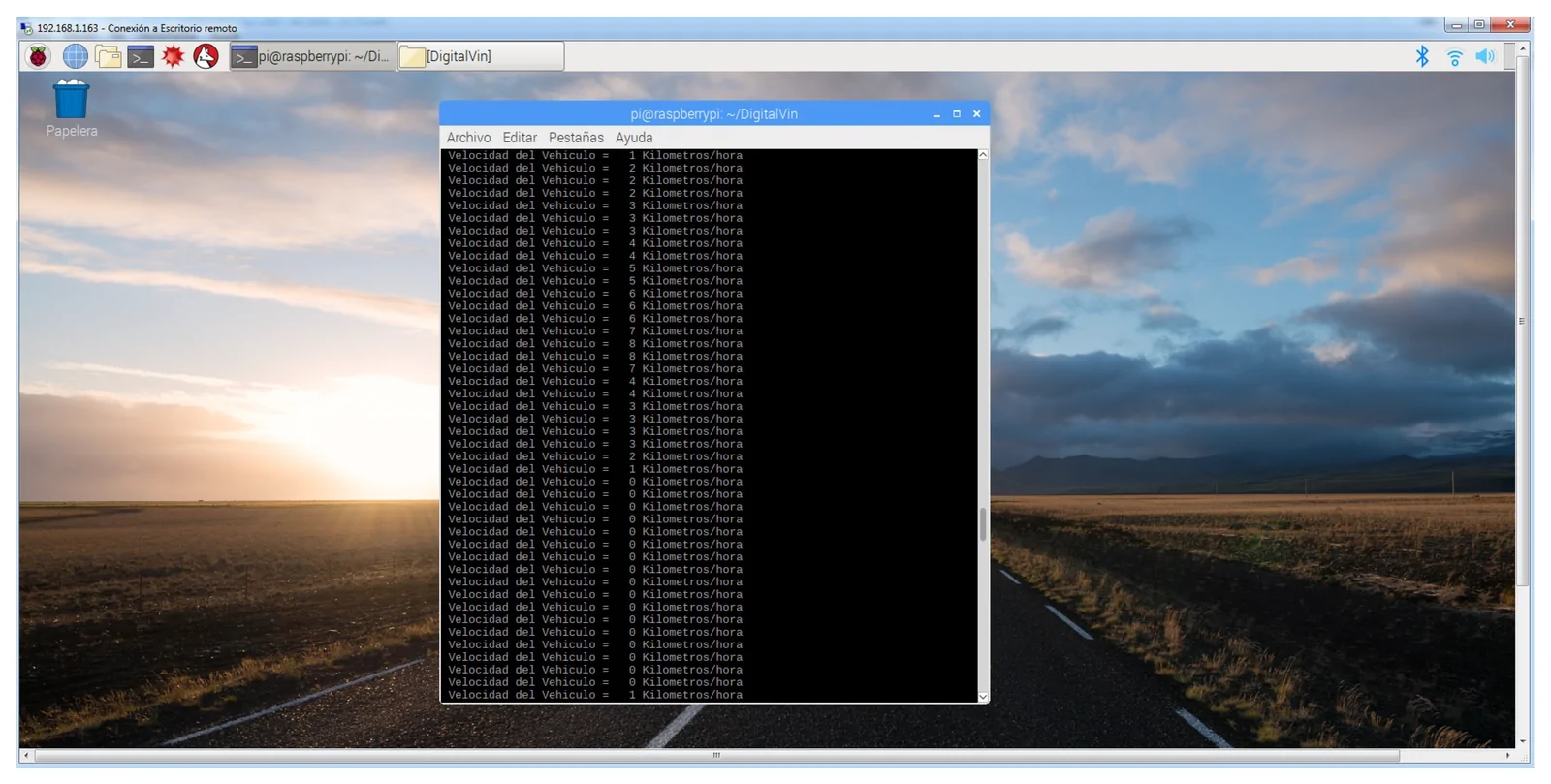
Speed value received [54].

**Figure 14:**
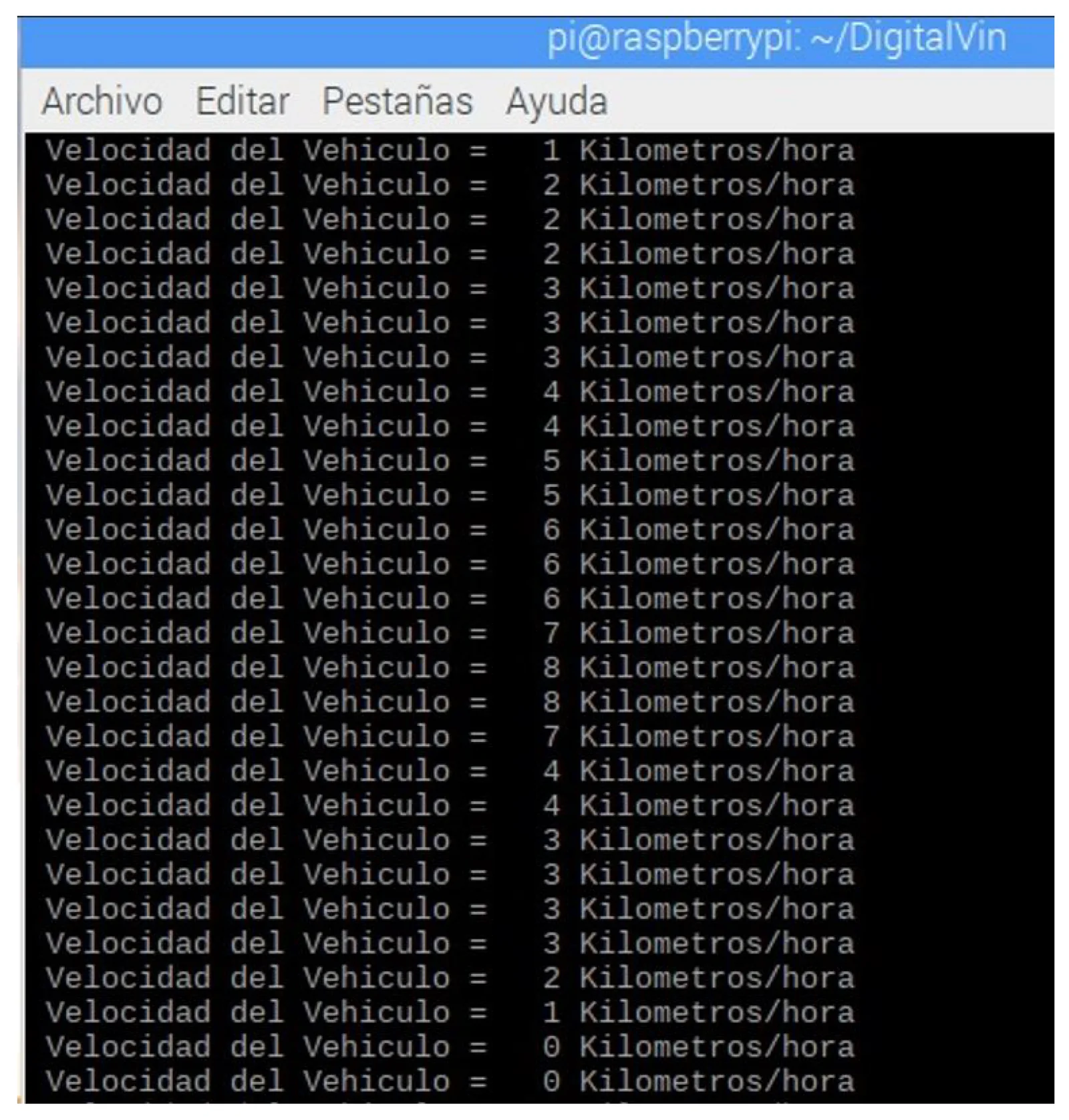
Detailed speed value received [54].

To develop our tests, we established communication between vehicles, enabling us to read the speed, direction angle, and GPS coordinates from the first car through its CAN bus. Then, we encrypted these data using the proposed protocol and using the VIN of the vehicles as the public keys corresponding to their identities. Afterwards, the second vehicle could process this and decrypt the information previously sent, as we can see in Figure 13 and Figure 14.

## 6. Conclusions

This paper proposes a communication protocol between vehicles using identity-based cryptography, eliminating the tedious task of creating and managing certificates typically required by PKIs. The protocol uses the vehicle’s digital VIN as its identity, representing a significant advance that ensures unequivocal identification comparable to traditional methods. This approach not only simplifies the authentication process, but also enables a wide range of applications in secure vehicular communications. A proof of concept was developed to demonstrate the efficiency and practicality of the proposed protocol. The experimental results showed low processing times and minimal computational overhead for both vehicles involved, which confirmed the suitability of the protocol for real-time vehicular environments. These findings highlight the ability of the protocol to provide secure and reliable communication, without compromising performance. In general, the results validated that the identity-based protocol can effectively address key challenges in V2V communication, such as reducing certificate management complexity while maintaining strong security guarantees. This work lays a solid foundation for future enhancements and potential deployment in intelligent transportation systems, contributing to safer and more efficient vehicular networks. The proposed system enables secure and versatile vehicle identification, with multiple practical applications. It can facilitate digital documentation for authentication with authorities and vehicle history tracking. It can support seamless communication for toll payments, without extra hardware, and can serve as a payment gateway at places like gas stations and kiosks. Using the VIN for V2X identification can enhance information exchange for traffic management and safety. Additionally, it can streamline processes at repair workshops by quickly transmitting vehicle and owner information, as well as improving fuel management and payment efficiency at gas stations.

## 7. Future Work

It would be interesting to develop mechanisms and tools to avoid VIN spoofing in the digital format. Similarly, as new network technologies such as 5G and 802.11p are deployed, it would be interesting to test using these standards, and ideally, the tests should be performed at higher speeds to demonstrate the robustness of the developed algorithm. Moreover, It will be important to study how the algorithms need be adapted to the new latency requirements established.

## Figures and Tables

**Table 1:** Comparison.

Technology	Cited References	Disadvantages	ID System
OCR	[15,29,30,31,32,36]	It is not reliable 100 × 100	Plates
GSM	[35,38]	Limited authentication	ICCID
RFID	[34,37,38]	Limited range	Random

**Table 2:** WiFi 802.11P Features.

Standard	WiFi Based on 802.11P
Range	From 100 to 1000 m
Band	5.9 GHz
Channels	Seven channels, each 10 MHz wide
Modulation	OFDM
Transfer rate	3, 4, 5, 6, 9, 12, 18, 24, and 25 Mbps in 10 MHz channels; up to 54 Mbps in 20 MHz channels
Subcarrier	52 subcarriers BPSK, QPSK, 16-QAM, or 64-QAM
Specific channels	Six for service and one for control

**Table 3:** 5G C-V2X Features.

Standard	3GPP 5G
Range	More than 1000 m
Bandwidth	700 MHz, 3,6, 26 GHz
Channels	100 MHz per operator
Modulation	OFDM
Transfer rate	100 Mbps, 1, 3, or 10 Gbps depending on the bandwidth

**Table 4:** Components used for the proof of concept in the first vehicle.

Type	Manufacturer	Model
Hardware	Raspberry Pi Foundation, Cambridge, UK	Pi 3
Hardware	SK Pang Electronics Ltd, Essex, UK	Skpang PiCAN
Hardware	Uputronics Ltd, Bingley, UK	Uputronics GPS/RTC
Operating System	Raspberry Pi Foundation	Raspbian Lite
Hardware	Oem	OBD2 interface
Vehicle	BMW AG, Munich, Germany	BMW X1 WBAVN71060VX14326

**Table 5:** Components used for the proof of concept in the second vehicle.

Type	Manufacturer	Model
Hardware	Vector Informatik GmbH, Stuttgart, Germany	CANalyze CIA DS102-2
Operating System	Microsoft Corporation, Redmond, WA, USA	Windows 10
Hardware	ASUSTeK Computer Inc., Taipei, Taiwan	F510D
Hardware	Oem	obd2 interface
Vehicle	BMW AG, Munich, Germany	BMW 320D WBAUT31010F150820

**Table 6:** Components, server, and WiFi.

Type	Manufacturer	Model
Hardware	ASUSTeK Computer Inc., Taipei, Taiwan	Asus Cloned PC i5-3470, 3.20 GHz, 16 Gb RAM, hard disk SSD
Operating System	Microsoft Corporation, Redmond, WA, USA	Windows 10
Software	VMware, Inc., Palo Alto, CA, USA	VMware Workstation Player
Hardware	TP-Link Technologies Co., Ltd., Shenzhen, China	Archer C6–AC1200

**Table 7:** Execution time at 0 km/h.

Vehicle Initiates	Vehicle Receives	Established Connection	Processing Time A (s)	Processing Time B (s)	Iteration
A	B	OK	0.0982	0.2812	1
A	B	OK	0.0987	0.2814	2
A	B	OK	0.0989	0.2821	3
A	B	OK	0.0995	0.2799	4
A	B	OK	0.0988	0.2788	5
A	B	OK	0.0983	0.2822	6
A	B	OK	0.0981	0.2718	7
A	B	OK	0.0986	0.2819	8
A	B	OK	0.0982	0.2787	9
A	B	OK	0.0983	0.2813	10

**Table 8:** Execution time at 5 km/h.

Vehicle Initiates	Vehicle Receives	Established Connection	Processing Time A (s)	Processing Time B (s)	Iteration
A	B	OK	0.0985	0.2842	1
A	B	OK	0.0991	0.2818	2
A	B	OK	0.0992	0.2832	3
A	B	OK	0.0996	0.2877	4
A	B	OK	0.1001	0.2901	5
A	B	OK	0.0982	0.2845	6
A	B	OK	0.0983	0.2721	7
A	B	OK	0.0988	0.2822	8
A	B	OK	0.0988	0.2791	9
A	B	OK	0.0987	0.2845	10

**Table 9:** Processing times in seconds at 0 km/h.

	A	B
Average	0.09856	0.27993
Variance	1.684 × 10^−7^	8.8281 × 10^−6^
Standard deviation	0.000410366	0.002971212

**Table 10:** Processing times in seconds at 5 km/h.

	A	B
Average	0.09893	0.28294
Variance	3.121 × 10^−7^	2.14544 × 10^−5^
Standard deviation	0.000558659	0.004631889

**Figure A1:**
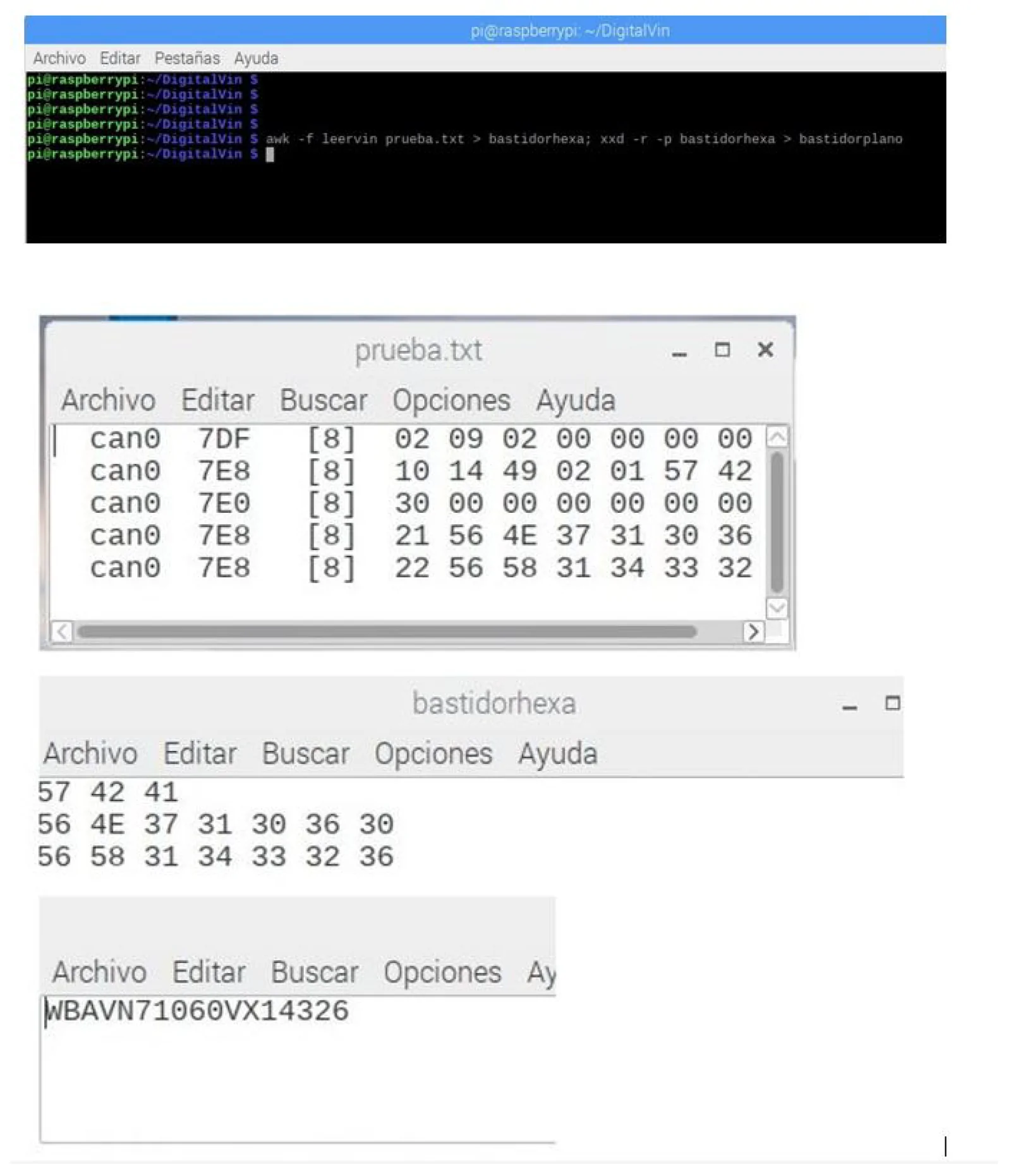
Digital VIN script.

## Data Availability

The original contributions presented in this study are included in the article. Further inquiries can be directed to the corresponding author.

## Abbreviations

The following abbreviations are used in this manuscript:

OBD2	On board diagnostics 2
ECU	Electronic control unit
IBC	Identity-based cryptography

## Appendix A. Fiore et Gennaro Protocol

**Set up:** The Key Generation Center (KGC) selects a finite group *G* of prime order *q*, a random generator of the group g∈G, and an exponent $x\in Z_{q}$. Two hash functions $H_{1}:{\left\{0,1\right\}}^{*}\to Z_{q}$ and $H_{2}:Z_{q}\times Z_{q}\to {\left\{0,1\right\}}^{\ell }$ are also selected. The public parameters are $MPK=(G,q,g,y=g^{x},H_{1},H_{2})$, and the master secret key will be the exponent *x*.

**Key Derivation:** To compute the private key of a user with identity ID, the KGC computes it as the Schnorr’s signature of the string ID under public key *y*, i.e., $r_{ID}=g^{k_{ID}}$, where $k_{ID}\in Z_{q}$ and $s_{ID}=k_{ID}+xH1\left(ID;r_{ID}\right)\;\mathrm{mod}\;q$.

**Key Agreement:** A and B choose ephemeral private exponents $t_{A},t_{B}\in Z_{q}$. They exchange the following messages:

$$\frac{A\;\;\;\;\;\;\;\;\;\;\;B}{t_{A},r_{A}\in Z_{q}\;\;\;\;\;\;t_{B},r_{B}\in Z_{q}}$$

$$\overset{\;\;\;A,\;r_{A},\;u_{A}=g^{t_{A}}\;\;\;}{\to }$$

$$\overset{\;\;\;B,\;r_{B},\;u_{B}=g^{t_{B}}\;\;\;}{\leftarrow }$$

$$\begin{array}{cc} z_{1}={\left(u_{B}r_{B}y^{H_{1}\left(B,r_{B}\right)}\right)}^{t_{A}+s_{A}} & z_{1}={\left(u_{A}r_{A}y^{H_{1}\left(A,r_{A}\right)}\right)}^{t_{B}+s_{B}}\\ z_{2}=u_{B}^{t_{A}} & z_{2}=u_{A}^{t_{B}} \end{array}$$

$$K_{AB}=H_{2}\left(z_{1},z_{2}\right)$$

## Appendix B. VIN Gathering

To obtain the VIN, we can send a broadcast message (0x7DF) with the following details: a parameter ID (PID) request (0x09) for the VIN (0x02), with a length of 2 bytes.

The VIN string has 17 characters, while the CAN data frame can only have 8 bytes. Therefore, the ECU will answer with a (0x7E8) message with the information (0x1014), indicating an extended message with a length of 0x14 = 20 bytes. To complete this information, the ECU waits for a second request ID (0x7E0) with data 0x30 followed by seven times 0x00. When the ECU receives the second set of data, it replies with two new 0x7E8 frames. The first byte is set to 0x21 and the second one is set to 0x22, with the first “2” indicating that there are two more data frames and the second character (1 or 2) representing the sequence number of the data frame. The information obtained is shown in hexadecimal.

In our case, the values (57 42 41 56 4E 37 31 30 36 30 56 58 31 34 33 32 36), when interpreted in ASCII, indicate that the VIN is WBAVN71060VX14326, which corresponds to the BMW brand and the X1 model. Figure A1 shows the process to obtain the VIN.

**Table A1:** VIN gathering through OBD2.

Interface	Identifier	Length	Data Frame
can0	7DF	8	02 09 02 00 00 00 00 00
can0	7E8	8	10 14 49 02 01 **57 42 41**
can0	7E0	8	30 00 00 00 00 00 00 00
can0	7E8	8	21 **56 4E 37 31 30 36 30**
can0	7E8	8	22 **56 58 31 34 33 32 36**

## References

[1] González L., Vaca M., Lattarulo R., Calvo I., Perez J., Ruiz A. (2018). Análisis de riesgos de ciberseguridad en arquitectura de vehículos automatizados. XXXIX Jornadas Autom..

[2] OMS (2017). 10 datos sobre la seguridad vial en el mundo. https://prevencionar.com/2018/03/15/10-datos-sobre-la-seguridad-vial-en-el-mundo/.

[3] OPS/OMS (2017). OMS estima que 7 millones de muertes ocurren cada año debido a la contaminación atmosférica. https://www.paho.org/hq/index.php?option=comcontentview=articleid=9406:2014-7-million-deaths-annually-linked-air-pollutionItemid=135lang=es.

[4] (2010). IEEE Standard for Information Technology—Telecommunications and Information Exchange Between Systems—Local and Metropolitan Area Networks—Specific Requirements—Part 11: Wireless LAN Medium Access Control (MAC) and Physical Layer (PHY) Specifications Amendment 6: Wireless Access in Vehicular Environments (WAVE).

[5] (2019). Intelligent Transport Systems (ITS); Vehicular Communications; Basic Set of Applications; Part 1: Functional Requirements.

[6] (2010). Intelligent Transport Systems (ITS); Communications Architecture.

[7] (2014). Intelligent Transport Systems—Communications Access for Land Mobiles (CALM)—Architecture.

[8] (2016). IEEE Standard for Wireless Access in Vehicular Environments—Security Services for Applications and Management Messages.

[9] (2016). IEEE Standard for Wireless Access in Vehicular Environments—Networking Services.

[10] (2020). Dedicated Short Range Communications (DSRC) Message Set Dictionary.

[11] (2019). Road Vehicles—Vehicle to Grid Communication Interface.

[12] (2020). Technical Specification for 5G Vehicle-to-Everything Communication.

[13] (2012). Overview of the Internet of Things.

[14] (2018). Road Vehicles—Functional Safety.

[15] Satsangi M., Yadav M., Sudhish P.S. License Plate Recognition: A Comparative Study on Thresholding OCR and Machine Learning Approaches. Proceedings of the 2018 International Conference on Bioinformatics and Systems Biology (BSB).

[16] Shamir A. (1984). Identity-based cryptosystems and signature schemes. Workshop on the Theory and Application of Cryptographic Techniques.

[17] Boneh D., Franklin M. (2003). Identity-Based encryption from the Weil pairing. SIAM J. Comput..

[18] Gentry C., Silverberg A. Hierarchical ID-based cryptography. Proceedings of the 8th International Conference on the Theory and Application of Cryptology and Information Security.

[19] Sakai R., Ohgishi K., Kasahara M. Cryptosystems based on pairing. Proceedings of the Symposium on Cryptography and Information Security.

[20] Boyd C., Choo K.R. Security of Two-Party Identity-Based Key Agreement. Proceedings of the First International Conference on Cryptology in Malaysia.

[21] Chen L., Cheng Z., Smart N.P. (2007). Identity-based key agreement protocols from pairings. Int. J. Inf. Secur..

[22] Fiore D., Gennaro R. (2010). Identity-based key exchange protocols without pairings. Transactions on Computational Science X.

[23] Peng C., Chen J., Zhou L., Choo K.R., He D. (2020). CsiIBS: A post-quantum identity-based signature scheme based on isogenies. J. Inf. Secur. Appl..

[24] Canbus (2015). What Is CAN Bus?. https://www.csselectronics.com/pages/can-bus-simple-intro-tutorial.

[25] SNS Telecom & IT (2019). The V2X (Vehicle-to-Everything) Communications Ecosystem: 2019–2030—Opportunities, Challenges, Strategies & Forecasts. https://www.snstelecom.com/v2x.

[26] Technologies K. (2016). Solutions for 802.11p Wireless Access in Vehicular Environments (WAVE) Measurements. https://innovation-destination.com/2018/01/29/adas-v2x-trumps-self-driving-2/.

[27] Naik G., Choudhury B., Park J.M. (2019). IEEE 802.11bd 5G NR V2X: Evolution of radio access technologies for V2X communications. IEEE Access.

[28] Feng Y., He D., Niu L., Yang M., Guan Y. (2017). The Overview of Chinese Cooperative Intelligent Transportation System Vehicular Communication Application Layer Specification and Data Exchange Standard 1. https://discovery.researcher.life/article/the-overview-of-chinese-cooperative-intelligent-transportation-system-vehicular-communication-application-layer-specification-and-data-exchange-standard/c07f45aa845c3590bd8e079c3a3c5149.

[29] Qadri M.T., Asif M. Automatic number plate recognition system for vehicle identification using optical character recognition. Proceedings of the 2009 International Conference on Education Technology and Computer.

[30] Johnson A.S., Bird B.M. Number-plate Matching for Automatic Vehicle Identification. Proceedings of the IEE Colloquium on Electronic Images and Image Processing in Security and Forensic Science.

[31] Lotufo R.A., Morgan A.D., Johnson A.S. Automatic Number-Plate Recognition. Proceedings of the IEE Colloquium on Image analysis for Transport Applications.

[32] Fahmy M.M.M. Automatic Number-plate Recognition: Neural Network Approach. Proceedings of the VNIS’94 Vehicle Navigation and Information System Conference.

[33] Changshui W., Ming Y., Yuanming G., Bin Q.Y. Analysis of Vehicle Handling and Stability in Frequency Domain Based on System Identification Method. Proceedings of the 2010 WASE International Conference on Information Engineering.

[34] Jeevagan N., Santosh P., Berlia R., Kandoi S. RFID based vehicle identification during collisions. Proceedings of the IEEE Global Humanitarian Technology Conference (GHTC 2014).

[35] Kohli S., Sharma M. Real-time Extraction and Transmission of Vehicle Registration using GSM transmission technique. Proceedings of the 2019 10th International Conference on Computing, Communication and Networking Technologies (ICCCNT).

[36] Groza B., Andreica T., Berdich A., Murvay P.S., Gurban E.H. (2020). Prestvo: Privacy enabled smartphone based access to vehicle on-board units. IEEE Access.

[37] Chithra V., Akash A., Rao K.N., Sivaram V., Tharun D., Naveen B.M. Density-based traffic control and stolen vehicle detection using Arduino and RFID. Proceedings of the 2023 Intelligent Computing and Control for Engineering and Business Systems (ICCEBS).

[38] Tang Z., He J., Zheng J. Cooperative vehicle identification for safe connected autonomous driving. Proceedings of the 2023 IEEE/CIC International Conference on Communications in China (ICCC Workshops).

[39] Wang S., Yang D., Sheng H., Shen J., Zhang Y., Ke W. (2024). A Blockchain-Enabled Distributed System for Trustworthy and Collaborative Intelligent Vehicle Re-Identification. IEEE Trans. Intell. Veh..

[40] Ma Y., Li X., Shi W., Cheng Q. (2024). STCLA: An Efficient Certificateless Authenticated Key Agreement Scheme for the Internet of Vehicles. IEEE Trans. Veh. Technol..

[41] Wang P., Gong C., Quan Y., Jin X., Du M., Wang L. Research on License Plate Recognition Method Based on LPRNet and Improved YOLOv8. Proceedings of the 2024 39th Youth Academic Annual Conference of Chinese Association of Automation (YAC).

[42] Vibin V., Sivraj P., Vanitha V. Implementation of In-Vehicle and V2V Communication with Basic Safety Message Format. Proceedings of the 2018 International Conference on Inventive Research in Computing Applications (ICIRCA).

[43] Wei S., Zou Y., Zhang X., Zhang T., Li X. (2019). An integrated longitudinal and lateral vehicle following control system with radar and vehicle-to-vehicle communication. IEEE Trans. Veh. Technol..

[44] Seat (2019). SEAT:CODE. https://code.seat/.

[45] Zhao S., Zhang T., Wu N., Ogai H., Tateno S. Vehicle to vehicle communication and platooning for EV with wireless sensor network. Proceedings of the 2015 54th Annual Conference of the Society of Instrument and Control Engineers of Japan (SICE).

[46] Hwang H.Y., Oh S.M., Shin J. CAN gateway for fast vehicle to vehicle (V2V) communication. Proceedings of the 2015 International Conference on Information and Communication Technology Convergence (ICTC).

[47] Shuhaimi N.I., Juhana T. Security in Vehicular Ad-Hoc Network with Identity-Based Cryptography Approach: A survey. Proceedings of the 2012 7th International Conference on Telecommunication Systems, Services and Applications (TSSA).

[48] Andreica T., Groza B. Secure V2V Communication with Identity-based Cryptography from License Plate Recognition. Proceedings of the Sixth International Conference on Internet of Things, Management and Security (IOTSMS).

[49] Al-shareeda M.A., Anbar M., Manickam S., Hasbullah I.H. (2020). An efficient identity-based conditional privacy-preserving authentication scheme for secure communication in a vehicular ad hoc network. Symmetry.

[50] Wasef A., Shen X. Efficient group signature scheme supporting batch verification for securing vehicular networks. Proceedings of the 2010 IEEE International Conference on Communications.

[51] Gopinath N., Narayanan K.L., Sageengrana S., Meenakshi M. HAA: A Novel Hybrid Authentication Scheme for VANETs Integrating Certificate-Based and Identity-Based Cryptography with Advanced Attack Detection. Proceedings of the 2025 International Conference on Computing and Communication Technologies (ICCCT).

[52] (2012). CAESAR: Competition for Authenticated Encryption: Security, Applicability, and Robustness. http://competitions.cr.yp.to/caesar.html.

[53] (2017). Vehículos de Carretera. Número de identificación de los vehículos (VIN). Contenido y Estructura.

[54] Vanier J. (2016). App-vin-reader. GitHub. https://github.com/carloop/app-vin-reader/blob/master/src/app-vin-reader.cpp.

[55] Zhong M. Research on vehicle identification method of Judicial Expertise based on deep learning algorithm. Proceedings of the 2025 5th International Symposium on Computer Technology and Information Science (ISCTIS).

[56] Gordón P.E. (2021). ES1249504 Número de bastidor/chasis de un automóvil en formato digital. https://patentscope.wipo.int/search/en/detail.jsf;jsessionid=EC3B477650F5D25814CA6F00BDC554B2.wapp2nA?docId=ES299105622&_cid=P20-KCVZ3H-27158-3.

[57] SAE International (2012). J1979: E/E Diagnostic Test Modes. https://www.sae.org/standards/content/j1979_201202/.

[58] (2016). Road Vehicles—Communication Between Vehicle and External Equipment for Emissions-Related Diagnostics—Part 3: Diagnostic Connector and Related Electrical Circuits: Specification and Use.

[59] Bellare M., Canetti R., Krawczyk H., Koblitz N. (1996). Keying hash functions for message authentication. Proceedings of the 16th Annual International Cryptology Conference.

[60] Rogaway P. Authenticated-encryption with associated-data. Proceedings of the 9th ACM Conference on Computer and Communications Security (CCS).

[61] Muñoz F., Porta J., Contreras M. (2013). Redes Ad-Hoc. http://profesores.elo.utfsm.cl/~agv/elo322/1s14/projects/reports/G16/Redes%20Ad-Hoc.pdf.

[62] Vinel A. (2012). 3GPP LTE versus IEEE 802.11p/WAVE: Which technology is able to support cooperative vehicular safety applications?. IEEE Wirel. Commun. Lett..

[63] Araniti G., Campolo C., Condoluci M., Iera A., Molinaro A. (2013). LTE for vehicular networking: A survey. IEEE Commun. Mag..

